# A fate-map for cranial sensory ganglia in the sea lamprey^☆^

**DOI:** 10.1016/j.ydbio.2013.10.021

**Published:** 2014-01-15

**Authors:** Melinda S. Modrell, Dorit Hockman, Benjamin Uy, David Buckley, Tatjana Sauka-Spengler, Marianne E. Bronner, Clare V.H. Baker

**Affiliations:** a Department of Physiology, Development and Neuroscience, University of Cambridge, Anatomy Building, Downing Street, Cambridge CB2 3DY, UK; b Division of Biology 139-74, California Institute of Technology, Pasadena, CA 91125, USA; c Departmento de Biodiversidad y Biología Evolutiva, Museo Nacional de Ciencias Naturales MNCN-CSIC, c/ José Gutiérrez Abascal 2, Madrid 28006, Spain

**Keywords:** Placodes, Neural crest, Ophthalmic trigeminal, Profundal, Maxillomandibular trigeminal, Pax3/7

## Abstract

Cranial neurogenic placodes and the neural crest make essential contributions to key adult characteristics of all vertebrates, including the paired peripheral sense organs and craniofacial skeleton. Neurogenic placode development has been extensively characterized in representative jawed vertebrates (gnathostomes) but not in jawless fishes (agnathans). Here, we use *in vivo* lineage tracing with DiI, together with neuronal differentiation markers, to establish the first detailed fate-map for placode-derived sensory neurons in a jawless fish, the sea lamprey *Petromyzon marinus*, and to confirm that neural crest cells in the lamprey contribute to the cranial sensory ganglia. We also show that a pan-Pax3/7 antibody labels ophthalmic trigeminal (opV, profundal) placode-derived but not maxillomandibular trigeminal (mmV) placode-derived neurons, mirroring the expression of gnathostome Pax3 and suggesting that Pax3 (and its single Pax3/7 lamprey ortholog) is a pan-vertebrate marker for opV placode-derived neurons. Unexpectedly, however, our data reveal that mmV neuron precursors are located in two separate domains at neurula stages, with opV neuron precursors sandwiched between them. The different branches of the mmV nerve are not comparable between lampreys and gnatho-stomes, and spatial segregation of mmV neuron precursor territories may be a derived feature of lampreys. Nevertheless, maxillary and mandibular neurons are spatially segregated within gnathostome mmV ganglia, suggesting that a more detailed investigation of gnathostome mmV placode development would be worthwhile. Overall, however, our results highlight the conservation of cranial peripheral sensory nervous system development across vertebrates, yielding insight into ancestral vertebrate traits.

## Highlights

• The first detailed fate-map for placode-derived sensory neurons in a jawless fish.
• Pax3 is a pan-vertebrate marker for ophthalmic trigeminal placode-derived neurons.
• Maxillomandibular trigeminal neuron precursors are located in two separate domains.
• Confirmation that lamprey neural crest cells contribute to cranial sensory ganglia.
• Results overall highlight conservation of cranial sensory nervous system development.

## Introduction

The neural crest (reviewed in Hall and Gillis, 2013, Prasad et al., 2012) and cranial neurogenic placodes (reviewed in Schlosser, 2006, Schlosser, 2010; Grocott et al., 2012; Graham and Shimeld, 2013) are transient, distinct embryonic cell populations whose derivatives include many key vertebrate characters, including the craniofacial skeleton and the paired peripheral sense organs. The conservation of the neural crest gene regulatory network across all vertebrates has been demonstrated by gene expression and morpholino-mediated functional knockdown studies in lampreys (Sauka-Spengler et al., 2007, Nikitina et al., 2008, Sauka-Spengler and Bronner-Fraser, 2008), which, together with hagfishes, are the only surviving representatives of the jawless fishes (agnathans). Lampreys and hagfishes – the cyclostomes (reviewed in Osório and Rétaux, 2008, Shimeld and Donoghue, 2012) – occupy a key phylogenetic position for understanding vertebrate development and evolution, since any traits shared by jawed vertebrates (gnathostomes) and one or both cyclostome lineages can parsimoniously be assumed to have been present in the vertebrate ancestor. Although cranial neurogenic placode development has been extensively studied in representative gnathostomes (reviewed in Schlosser, 2006, Schlosser, 2010, Grocott et al., 2012, Graham and Shimeld, 2013), relatively little information is available in lampreys (*e.g.* von Kupffer, 1895, Damas, 1944, Fisk, 1954, Murakami et al., 2001, Neidert et al., 2001, McCauley and Bronner-Fraser, 2002).

In gnathostomes, all cranial neurogenic placodes originate from a specialized region of ectoderm around the anterior neural plate called the “pan-placodal primordium” or “preplacodal region” (reviewed in Streit, 2007, Ladher et al., 2010, Schlosser, 2010, Grocott et al., 2012). Within the developing preplacodal region, the rostral-caudal expression of mutually repressive members of the Pax family of paired domain transcription factors seems to be essential for regional identity and subsequent development of individual placodes: Pax6 for the “anterior” placodes (adenohypophyseal, olfactory, lens); Pax2/5/8 for the “posterior” placodes (otic, lateral line, epibranchial); and Pax3 for the “intermediate” placodes (trigeminal) (reviewed in Schlosser, 2010; Grocott et al., 2012). Similar *Pax* family gene expression patterns have been reported for lamprey placodes (Murakami et al., 2001, McCauley and Bronner-Fraser, 2002, Osório et al., 2005). However, the existence of two molecularly distinct trigeminal placodes is sometimes overlooked: in birds and mammals, Pax3 is expressed by and required for the differentiation of the ophthalmic trigeminal (opV) placode and opV placode-derived neurons in the ophthalmic lobe of the trigeminal ganglion (Stark et al., 1997, Baker et al., 1999, Xu et al., 2008, Dude et al., 2009), while the Pax3-negative maxillomandibular trigeminal (mmV) placode gives rise to Pax3-negative neurons in the maxillomandibular lobe of the same ganglion (D'Amico-Martel, 1982, D'Amico-Martel and Noden, 1983, Xu et al., 2008).

In lampreys, as in gnathostomes, the ophthalmic trigeminal (opV, V1) nerve transmits somatosensory information from the rostral part of the head, while the maxillomandibular trigeminal (mmV, V2/3) nerve performs the same function for the upper and lower lips and velum (see Kuratani et al., 1997, Kuratani et al., 2004, Murakami and Watanabe, 2009, Oisi et al., 2013). In anamniotes, separate “profundal” and “trigeminal” ganglia (fused in some groups) have been described, but *Pax3* expression in the profundal placode in representatives of the three major gnathostome lineages (cartilaginous fishes, and lobe-finned and ray-finned bony fishes) confirms the previously proposed hypothesis that the anamniote profundal placode and ganglion are homologous, respectively, with the amniote opV placode and the ophthalmic lobe of the amniote trigeminal ganglion (O'Neill et al., 2007, Schlosser and Ahrens, 2004, Modrell et al., 2011). OpV and mmV placodes have been described in lampreys (von Kupffer, 1895, Damas, 1944, Fisk, 1954), but it remains unclear whether these placodes (or the neurons derived from them) can be distinguished via Pax3 expression, as would be expected given the assumed homology of cyclostome and gnathostome opV/profundal ganglia (Northcutt, 1979, Koyama et al., 1987, Wicht and Northcutt, 1995, Kuratani et al., 1997, Kuratani et al., 2004, Murakami and Watanabe, 2009). To date, a single *Pax3/7* subfamily gene has been isolated from three lamprey species: an apparent *Pax7* ortholog in the sea lamprey *Petromyzon marinus* (McCauley and Bronner-Fraser, 2002) (also see O'Neill et al. (2007)), and an unresolvable *Pax3*/*7* gene in both the river lamprey *Lampetra fluviatilis* (Osório et al., 2005) and the Arctic lamprey *Lethenteron camtschaticum* (junior synonym *Lethenteron japonicum*) (Kusakabe et al., 2011). Similarly, a single *Pax3/7* gene was reported in the inshore hagfish *Eptatretus burgeri* (Ota et al., 2007). Although *Pax3/7* expression was described in the lamprey “trigeminal” placode and/or ganglion (McCauley and Bronner-Fraser, 2002, Osório et al., 2005), no distinction was made between opV and mmV placodes/ganglia.

Here, we have used neuronal differentiation markers and DiI labeling to construct the first detailed fate-map of neurogenic placodes in an agnathan, the sea lamprey *P. marinus*. In addition, after labeling premigratory neural crest cells up to a day earlier than in a previous study (McCauley and Bronner-Fraser, 2003), we show that cranial sensory ganglia in the sea lamprey also contain neural crest-derived cells. Our results suggest that the development of neurogenic placodes and cranial sensory ganglia is in general highly conserved across all vertebrates, including expression in the opV placode and opV placode-derived neurons of the single Pax3/7 ortholog in lampreys and Pax3 in gnathostomes. Unexpectedly, however, our data suggest that upper lip-innervating and lower lip/velum-innervating mmV neurons, which are spatially segregated within the lamprey mmV ganglion (Koyama et al., 1987, Murakami and Kuratani, 2008), may originate from spatially segregated precursors, with opV neuron precursors sandwiched between the two. Although this may be a derived feature of lampreys, maxillary and mandibular trigeminal neurons are spatially segregated in the gnathostome mmV ganglion, suggesting that more detailed investigation of the mmV placode could reveal spatial segregation of maxillary and mandibular trigeminal neuron precursors in gnathostomes.

## Materials and methods

### Embryo collection

*P. marinus* eggs were collected from adults and fertilized as described (Nikitina et al., 2009). Embryos were maintained at 18 °C in 0.1× or 1× Marc's modified Ringer's (MMR) solution.

### Phylogenetic analyses

To analyze the orthologous/paralogous relationships of the Pax3/7 family of transcription factors in chordates, phylogenetic analyses were performed under the Bayesian and coalescence-based frameworks using amino acid sequences available from GenBank (National Center for Biotechnology Information), Ensembl (http://www.ensembl.org) or SkateBase (http://www.skatebase.org; Wang et al., 2012). Detailed methodologies and a table of species names and accession numbers are available in Supplemental materials.

### DiI labeling

DiI labeling was performed as described (Nikitina et al., 2009), with some modifications. Briefly, embryonic day (E) 5–7 embryos (Piavis stages 11–12: late neurula) (Piavis, 1961, Richardson and Wright, 2003) were manually dechorionated in 0.1× MMR, then immobilized and oriented in 1× MMR in 18-mm Petri dishes that were either agarose-coated with depressions or lined with a fine mesh. Embryos were pressure-injected using glass capillary tubes filled with 0.5 mg/ml of Cell Tracker-CM-DiI (Invitrogen) diluted in 0.3 M sucrose (from a 5 µg/µl stock diluted in ethanol). They were allowed to recover in 1× MMR for 24 h, then individually transferred to an uncoated Petri dish containing 0.1× MMR and allowed to develop to E16–21 (Piavis stages 15–17: *i.e.*, from embryos with a full complement of pharyngeal pouches, through to embryos with open gill slits and eyespots) (Piavis, 1961, Richardson and Wright, 2003). Embryos were periodically checked and imaged throughout, then fixed in 4% paraformaldehyde in phosphate-buffered saline (PBS) for 1 h at room temperature.

### Generation of the fate-map

Individual images (taken at the same magnification) were superimposed onto template embryos at E6–7 and E20–21. Using Adobe Illustrator, DiI-labeled regions were outlined onto the template. Maps combining all labeled regions were generated for each placode and its associated ganglion, or for a combination of placodes and ganglia.

### Immunohistochemistry

Immunostaining was performed as described (Nikitina et al., 2009) with slight modifications; embryos were incubated overnight at 4 °C in primary antibody in blocking solution (10% sheep serum in PBS with 0.1% Triton X-100); secondary antibodies were also incubated overnight at 4 °C. Histochemical reactions were performed as described (Patel, 1994). Before imaging, embryos were cleared through a glycerol series into 70% glycerol in PBS. Primary antibodies: 1:50 HNK1 (mouse IgM, clone 3H5, Developmental Studies Hybridoma Bank); 1:500 anti-HuC/D (mouse IgG2b; Invitrogen); 1:200 anti-neurofilament-M (mouse IgG2a; Invitrogen); 1:200 anti-Pax3/7 (clone DP312; Davis et al., 2005). (The Developmental Studies Hybridoma Bank was developed under the auspices of the NICHD and is maintained by the University of Iowa, Department of Biological Sciences, Iowa City.) Secondary antibodies: 1:1000 Alexa^488^-conjugated goat anti-mouse IgG and/or Alexa^594^-conjugated goat anti-mouse IgG (Invitrogen), or 1:600 horseradish peroxidase-conjugated or alkaline phosphatase-conjugated goat anti-mouse IgG (Jackson ImmunoResearch).

### Histology

For cryosectioning, embryos were incubated in PBS with 5% sucrose for 4 h at room temperature. After overnight incubation at 4 °C in 15% sucrose in PBS, they were transferred into prewarmed 7.5% gelatin in 15% sucrose in PBS and incubated for 1–4 h at 37 °C, then oriented and embedded in molds, frozen by immersion in liquid nitrogen or a dry ice-isopentane solution for 30 s, and cryosectioned at 12–16 µm. Gelatin was removed by a 5-min incubation in PBS prewarmed to 37 °C. For paraffin wax sectioning, embryos were dehydrated into 100% methanol, cleared by step-wise transfer into Histosol (National Diagnostics), embedded by step-wise transfer into Paraplast (Fisher Scientific) at 60 °C, and sectioned at 8–12 µm using a rotary microtome. Slides were de-waxed in Histosol and rehydrated into PBS through a graded ethanol series. After immunostaining, sections were counterstained with the nuclear marker DAPI (1 ng/ml) (Invitrogen) and mounted in Fluoromount G (Southern Biotech).

## Results

### Development of cranial sensory ganglia in the sea lamprey

**Fig. 1:**
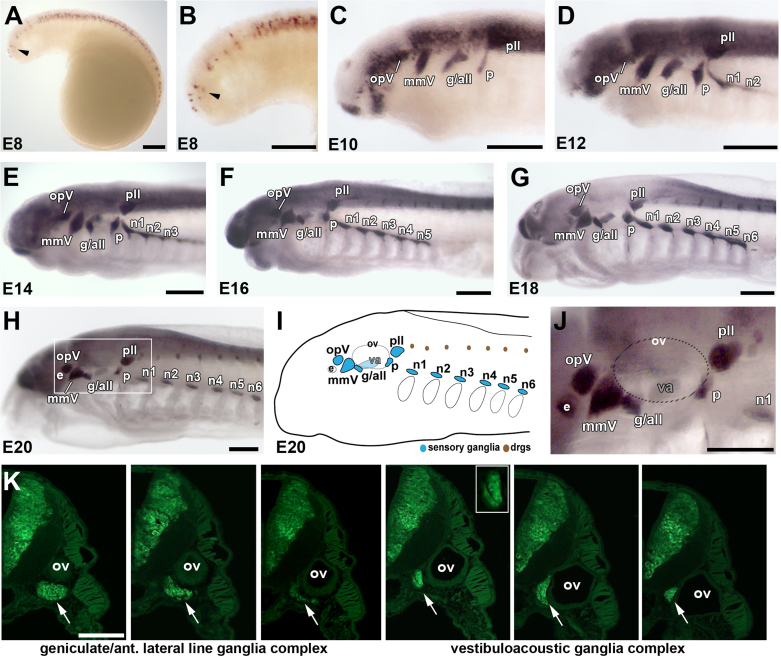
Spatiotemporal development of lamprey cranial sensory ganglia. Anterior is to the left for all whole-mount images. (A) and (B) Low-power (A) and higher-power view (B) of an embryo at embryonic day 8 (E8) immunostained for the pan-neuronal Elav-family members HuC/D (Hu). HuC/D expression is strong in neurons within the neural tube, with fainter expression in neurons lateral to the rostral neural tube (arrowhead). (C) By E10, discrete lateral patches of HuC/D expression reveal the primordia of all cranial sensory ganglia except the nodose: the ophthalmic trigeminal ganglion (opV), the maxillomandibular trigeminal ganglion (mmV), the geniculate/anterior lateral line ganglionic complex (g/all), the petrosal ganglion (p) and the posterior lateral line ganglion (pll). (D)–(G) HuC/D immunostaining of embryos at E12 (D), E14 (E), E16 (F) and E18 (G) shows the development of the six nodose ganglia in a rostrocaudal sequence dorsal to the branchial arches and the progressive condensation of all the cranial ganglia. Dorsal root ganglia are also visible from E16, adjacent to the dorsal neural tube. (H)–(J) By E20, all the cranial sensory ganglia have formed. (H) Low-power and (I) schematic view of an E20 embryo, showing the location of cranial sensory ganglia [blue in (I)] and dorsal root ganglia [brown in (I)]. (J) A higher-power view of the boxed area in (H), showing distinct opV and mmV ganglia, the geniculate/anterior lateral line ganglionic complex, the vestibuloacoustic ganglion (medial to the otic vesicle, hence hardly stained in whole-mount), the petrosal ganglion, the posterior lateral line ganglion and the most rostral nodose ganglion (n1). (K) Transverse serial sections immunostained for HuC/D (green), starting near the rostral edge of the otic vesicle [see panel (J) for orientation of otic vesicle, which is indicated by a dotted oval] and progressing caudally through the geniculate/aLL ganglionic complex ventral to the otic vesicle (arrow, left-hand three images) and then the vestibuloacoustic ganglion medial to the otic vesicle (arrow, right-hand three images). In the fourth and fifth images, the developing intracapsular ganglion (second ganglion of the anterior lateral line nerve) may also be visible, medial to the vestibuloacoustic ganglion and slightly separated from it by a thin HuC/D-negative space (inset). Abbreviations: all, anterior lateral line ganglion; drgs, dorsal root ganglia; e, eye; g, geniculate ganglion; mmV, maxillomandibular trigeminal ganglion; n, nodose ganglion; opV, ophthalmic trigeminal (profundal) ganglion; ov, otic vesicle; p, petrosal ganglion; pll, posterior lateral line ganglion; va, vestibuloacoustic ganglion. Scale bars: (A)–(J) 0.2 mm; (K) 50 μm.

The developing cranial sensory ganglia in *P. marinus* embryos were visualized by whole-mount immunostaining for the neuronal Elav RNA-binding protein family members HuC/D (Hinman and Lou, 2008) (Fig. 1) and identified according to established descriptions of neurogenic placode and cranial ganglion development in the European brook lamprey *Lampetra planeri* (also referred to as *P. planeri*, *Ammocoetes planeri*) (von Kupffer, 1891, von Kupffer, 1895, Fisk, 1954), the river lamprey *Lampetra fluviatilis* (Damas, 1944) and the Arctic lamprey *Lethenteron camtschaticum* (*Lethenteron* is a subgenus of *Lampetra*; junior synonyms include *Lethenteron japonicum* and *Lampetra japonica*) (Kuratani et al., 1997, Murakami and Watanabe, 2009). Starting at embryonic day (E) 8 (Piavis stage 12/13; Piavis, 1961, Richardson and Wright, 2003), HuC/D was observed in the neural tube, and more weakly, in presumptive opV and/or mmV placode-derived neurons (Fig. 1A and B). By E10 (Piavis stage 14), HuC/D expression revealed the separate opV and mmV ganglia; the small presumptive anterior lateral line (aLL) ganglion lying immediately dorsocaudal to the geniculate ganglion (*i.e.*, the first epibranchial placode-derived ganglion, dorsal to the first pharyngeal pouch); and the very large posterior lateral line (pLL) ganglion lying immediately dorsocaudal to the petrosal ganglion (*i.e.*, the second epibranchial placode-derived ganglion, dorsal to the second pharyngeal pouch) (Fig. 1C). Fig. 1D–H show the further development of the cranial sensory ganglia between E12 and E20 (Piavis stages 14–17), now including the developing chain of nodose ganglia (*i.e.*, the third and more caudal epibranchial placode-derived ganglia, which form dorsal to the third and more caudal pharyngeal pouches), as well as dorsal root ganglia (Fig. 1F–H; compare with Figs. 7a and 8a in Kuratani et al., 1997). By E20 (Fig. 1H–J), almost all cranial sensory ganglia could be distinguished except the vestibuloacoustic ganglion (also unidentified in Kuratani et al., 1997), which lies medial to the otic vesicle. The whole-mount HuC/D immunostaining data at E20 are summarized in schematic form in Fig. 1I. HuC/D immunostaining on transverse serial sections (Fig. 1K) confirmed the presence of a large, seemingly contiguous ganglionic complex extending rostral and medial to the otic vesicle. This complex most likely comprises the fused geniculate and aLL ganglia, followed by the vestibulo-acoustic ganglion medial to the otic vesicle and perhaps also the even more medial intracapsular ganglion (*i.e.*, the second ganglion of the aLL nerve; Koyama et al., 1990), which in the adult lamprey is located within the otic capsule, immediately medial to the vestibuloacoustic ganglion (Koyama et al., 1990).

### A pan-Pax3/7 antibody labels opV placode-derived neurons

**Fig. 2:**
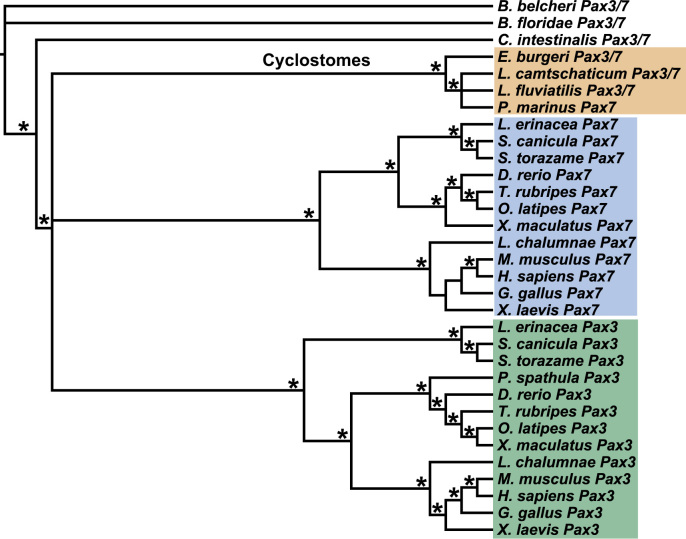
Cyclostome Pax3/7 subfamily proteins form a well-supported clade, separate from gnathostome Pax3 and Pax7 clades. Maximum *a posteriori* (MAP) topology, obtained with Bali-Phy software, summarizing all of the phylogenetic analyses performed. Nodes consistently recovered with high support in all Bayesian and coalescence-based analyses are indicated by an asterisk (where the posterior probability is >90). These analyses consistently recovered three main clades of orthologous sequences: (i) a cyclostome clade containing all published hagfish and lamprey Pax3/7 proteins (orange box), (ii) a gnathostome Pax7 clade (blue box) and (iii) a gnathostome Pax3 clade (green box). Relationships between these three clades, however, remain unresolved: the gnathostome Pax3 and Pax7 clades formed a sister group in most analyses but with low support. Within the gnathostome clades, relationships are well resolved for the Pax3 but not the Pax7 subfamily: in some cases, short sequence lengths for Pax7 likely account for a topology that is incongruent with the currently proposed relationships among vertebrates. Generally, however, phylogenetic relationships recovered here are consistent with current understanding of the chordate phylogeny.

During neurogenic placode development in gnathostomes, only the opV placode and opV placode-derived neurons express Pax3 (Stark et al., 1997, Baker et al., 1999, Schlosser and Ahrens, 2004, O'Neill et al., 2007, Modrell et al., 2011), which is required for opV placode development and opV neuron differentiation (Dude et al., 2009). Detailed phylogenetic analyses of the Pax3/7 subfamily of transcription factors, which included Pax3/7 protein sequences from one hagfish (Ota et al., 2007) and three lamprey species (McCauley and Bronner-Fraser, 2002, Osório et al., 2005, Kusakabe et al., 2011), showed a well-supported cyclostome Pax3/7 clade and separate gnathostome Pax3 and Pax7 clades (Fig. 2). However, the relationships between the cyclostome Pax3/7 clade and the gnathostome Pax3 and Pax7 clades remained unresolved in these analyses, resulting in a polytomy (Fig. 2).

**Fig. 3:**
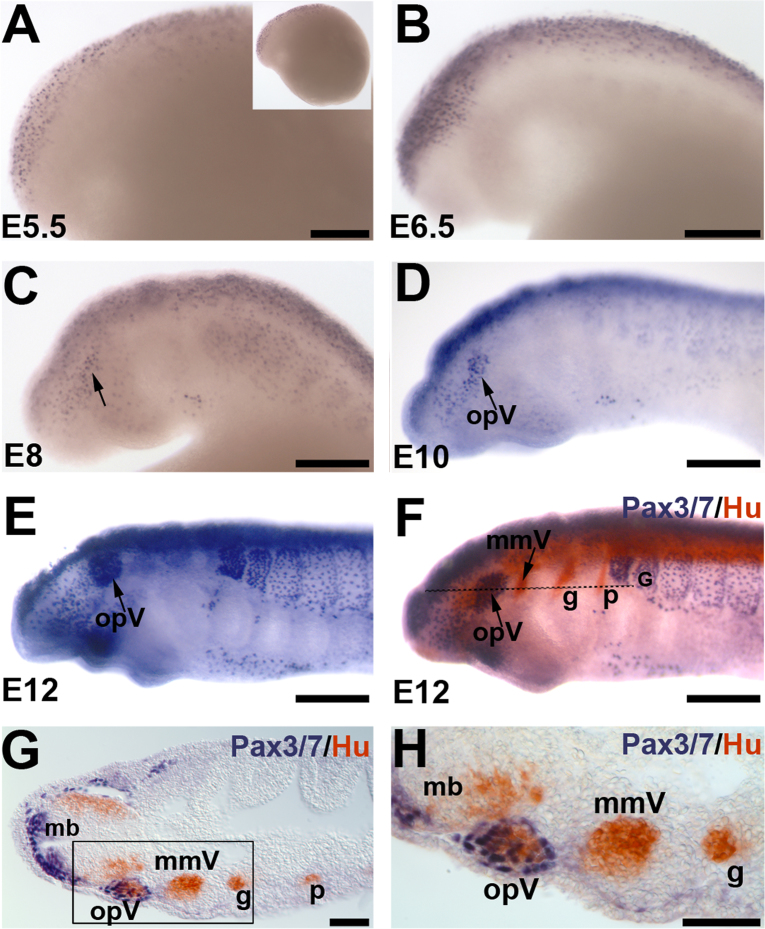
Pax3/7 is a specific marker for opV placode-derived neurons. Whole-mount immunostaining with the pan-Pax3/7 antibody DP312 (Davis et al., 2005) shows (A) Pax3/7 expression at E5.5 in the anterior-most dorsal neural tube; inset shows entire embryo. (B) By E6.5, more Pax3/7-positive cells appear along the length of the neural tube and in presumptive migrating neural crest cells adjacent to it. (C) At E8, Pax3/7 expression is seen in the neural tube, somites and scattered cells lateral to the neural tube (arrow). (D) By E10, a patch of Pax3/7-positive cells (arrow) is seen lateral to the neural tube in a similar position to the developing opV ganglion (compare with Fig. 1C). (E) By E12, Pax3/7 is strongly expressed in the presumptive opV placode/ganglion (opV, arrow); (F) this is confirmed by double immunostaining for Pax3/7 (blue) and the pan-neuronal marker HuC/D (red). Dotted line shows plane of section in (G) and (H). (G) Coronal section shows Pax3/7 expression in the dorsal neural tube and opV ganglion, but not in the mmV, geniculate or petrosal ganglia. (H) A higher-power view of the boxed region in (E) confirms that Pax3/7 expression is restricted to the developing opV ganglion (and dorsal neural tube cells). Abbreviations: g, geniculate ganglion; mb, midbrain; mmV, maxillomandibular trigeminal ganglion; opV, ophthalmic trigeminal placode/ganglion; p, petrosal ganglion. Scale bars: (A)–(F) 0.2 mm; (G) and (H) 50 μm.

We wished to determine whether lamprey Pax3/7, like gnathostome Pax3, is expressed by opV but not mmV placode-derived neurons. To address this question, we used a cross-reactive Pax3/7 antibody (Davis et al., 2005) to immunostain *P. marinus* embryos in whole-mount. This antibody has demonstrated broad species cross-reactivity to Pax3/7 proteins in arthropods, invertebrate chordates and vertebrates (Davis et al., 2005, Somorjai et al., 2012, Minchin and Hughes, 2008, Curran et al., 2010) and its core epitope, PD(V/I)YTREE (Davis et al., 2005), is present in the homeodomain of the *P. marinus* Pax3/7 protein (McCauley and Bronner-Fraser, 2002). At E5.5, Pax3/7-positive cells were observed primarily in dorsal regions of the developing brain (Fig. 3A). By E6.5, stronger Pax3/7 expression was found along the dorsal neural tube and also adjacent to it, presumably in migrating neural crest cells (Fig. 3B). Between E8 and E10, a discrete patch of Pax3/7-positive cells appeared in a pattern and location similar to the opV ganglion (Fig. 3C and D; compare with Fig. 1A and B). By E12, this patch strongly expressed Pax3/7 (Fig. 3E). Double immunostaining for Pax3/7 and HuC/D in whole-mount (Fig. 3F), followed by coronal sectioning (Fig. 3G and H), confirmed that Pax3/7 expression was restricted to the developing opV ganglion and excluded from the mmV ganglion. Taken together with information on gnathostome Pax3 expression (Stark et al., 1997, Baker et al., 1999, Schlosser and Ahrens, 2004, O'Neill et al., 2007, Modrell et al., 2011) and function (Dude et al., 2009), these data support an evolutionarily conserved role for Pax3 in patterning the opV placode and ganglion in all vertebrates.

### OpV neuron precursors are initially sandwiched between two separate domains of mmV neuron precursors

**Fig. 4:**
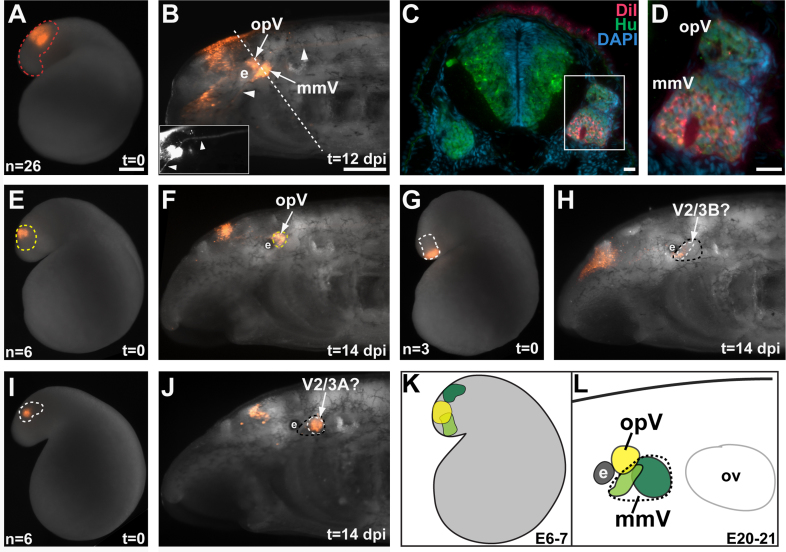
Fate-mapping of opV and mmV placode-derived neurons reveals two separate domains of mmV neuron precursors at late neurula stages. Anterior is to the left for all whole-mount images. (A) An E6.5 embryo immediately after DiI injection into a broad patch of anterodorsal head ectoderm within the region outlined in red, which contains both opV and mmV neuron precursors. (B) The same embryo as in (A) at E18 (*t*=12 dpi), showing DiI labeling in the opV and mmV ganglia. DiI is also visible in the upper lip-innervating mmV nerve (V2/3A, inset, ventral arrowhead) and central projections from the mmV ganglion (inset, dorsal arrowhead). Dotted line shows plane of section in (C) and (D). (C) Merged images of low-power and (D) higher-power views of an oblique section through both the opV and mmV ganglia, immunostained for the neuronal marker HuC/D (green) and counterstained for the nuclear marker DAPI (blue), showing DiI (red) in surface ectoderm and also co-localized with neurons (HuC/D, green) in both the opV and mmV ganglia. (E) An E6.5 embryo immediately after focal DiI injection into the region of head ectoderm outlined in yellow, which contains opV neuron precursors. (F) The same embryo as in (E) at E20 (*t*=14 dpi), showing restriction of DiI to the opV ganglion. (G) An E6.5 embryo immediately after a focal DiI injection into the region of head ectoderm outlined in white (shaded light green in schematic). (H) The same embryo as in (G) at E20 (*t*=14 dpi), showing DiI localization to a relatively small, rostral domain (outlined in white) of the mmV ganglion (outlined in black), which may correspond to lower lip/velum-innervating V2/3B neurons (see text). (I) An E6.5 embryo immediately after a focal DiI injection into the region of head ectoderm outlined in white (shaded dark green in schematic). (J) The same embryo as in (I) at E20 (*t*=14 dpi), showing DiI localization to a larger, caudal domain (outlined in white) of the mmV ganglion (outlined in black), which may correspond to upper lip-innervating V2/3A neurons (see text). (K) Schematic fate-map at E6–7 summarizing the location of opV neuron precursors (yellow) between two separate patches of mmV neuron precursors (light and dark green). (L) Schematic summarizing the fate at E20–21 of DiI-injected cells within the locations shown in panel (K). The opV ganglion (yellow) lies dorsal to the mmV ganglion (dotted black outline). Rostral (light green) and caudal (dark green) subregions of the mmV ganglion are distinguishable in the E6–7 fate-map, which may correspond to V2/3B and V2/3A neurons, respectively (see text). Abbreviations: dpi, days post-injection; e, eye; mmV, maxillomandibular trigeminal ganglion; opV, ophthalmic trigeminal ganglion; ov, otic vesicle; t, time, V2/3A, upper lip-innervating trigeminal neurons; V2/3B, lower lip/velum-innervating trigeminal neurons. Scale bars: (A), (E), (G) and (I) 0.2 mm; (B), (F), (H) and (J) 0.2 mm; (C) and (D) 10 μm.

The maxillary and mandibular branches of the mmV (V2/3) nerve are not comparable between lampreys and gnathostomes (see Kuratani et al., 1997, Kuratani et al., 2004, Shigetani et al., 2002, Murakami and Watanabe, 2009, Oisi et al., 2013), hence we have followed here the nomenclature proposed by Oisi et al. (2013) (see Supplementary Fig. 8 in Oisi et al., 2013), in which “V2/3A” designates the upper lip-innervating anterior branch and “V2/3B” the lower lip/velum-innervating posterior branch of the lamprey mmV nerve (see Kuratani et al., 1997, Murakami and Watanabe, 2009, Oisi et al., 2013). We used the vital lipophilic dye DiI to label discrete regions of cranial ectoderm at E6–7 (late neurula; Piavis stages 11–12), and followed subsequent development for 12–14 days, to approximately E18–21 (Piavis stage 17). In embryos in which DiI was injected into a broad patch of anterodorsal head ectoderm, represented by the red dotted line in Fig. 4A, DiI was observed in the condensing opV and mmV ganglia by 12 days post-injection (dpi) (E18–19) (*n*=26; Fig. 4B; compare with Fig. 1G and H). Furthermore, ophthalmic (V1) and upper lip-innervating (V2/3A) nerve branches, originating respectively from the opV and mmV ganglia, were also labeled with DiI (Fig. 4B arrows and inset). After sectioning in an oblique plane to include both ganglia, HuC/D immunostaining confirmed that the DiI-positive cells were located in the opV and mmV ganglia (Fig. 4C and D).

More focal DiI injections at E6–7 within this broader region allowed us to distinguish the location of opV and mmV neuron precursors. After DiI injection into a roughly central sub-region of the broader domain, outlined with the yellow dotted line in Fig. 4E (compare with the broader domain outlined in red in Fig. 4A), DiI-labeled cells were later observed only in the opV ganglion (*n*=6; Fig. 4F; schematized in yellow in Fig. 4K and L). To our surprise, mmV neuron precursors were found at E6–7 in two spatially segregated domains, correlating with the position of labeled neurons within the mmV ganglion at E20–21. A region of ectoderm anteroventral to (but partially overlapping with) the patch of opV neuron precursors gave rise to neurons in a relatively small, rostral domain of the mmV ganglion at E20–21, potentially corresponding to lower lip/velum-innervating V2/3B neurons (Kuratani et al., 2004, Murakami and Kuratani, 2008) (*n*=3; Fig. 4G and H; schematized in light green in Fig. 4K and L). In contrast, a region of ectoderm caudal to the patch of opV neuron precursors gave rise to neurons in a larger, caudal domain of the mmV ganglion at E20–21, potentially corresponding to upper lip-innervating V2/3A neurons (Kuratani et al., 2004, Murakami and Kuratani, 2008) (*n*=6; Fig. 4I and J; schematized in dark green in Fig. 4K and L).

### Development of epibranchial and lateral line ganglia

**Fig. 5:**
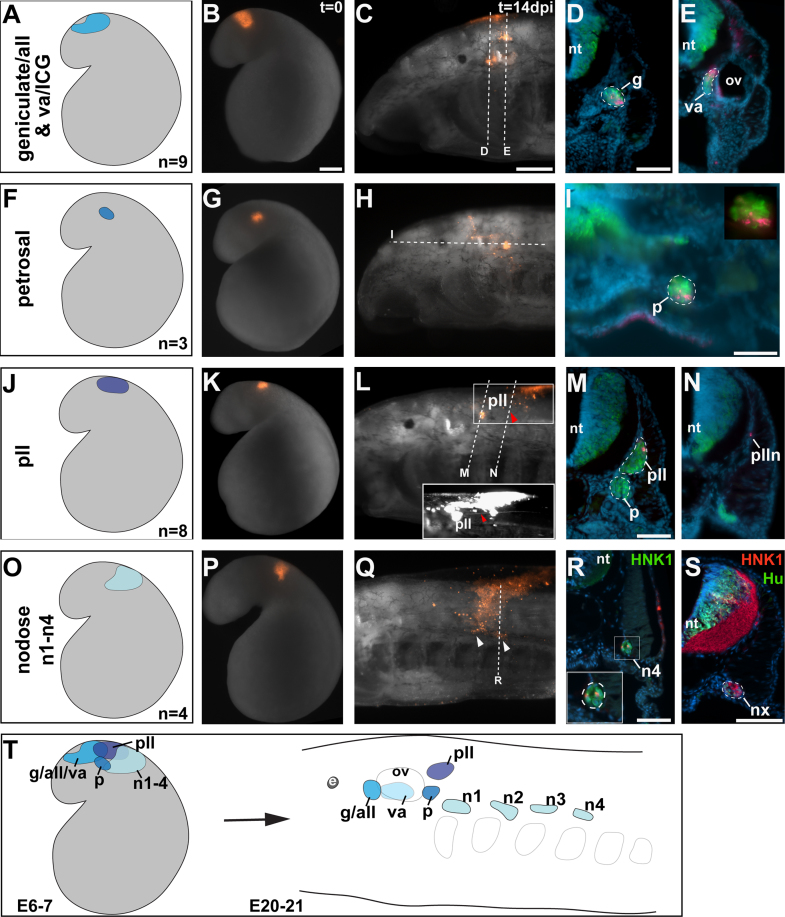
Fate-maps at E6-7 for lamprey epibranchial and lateral line placode-derived ganglia. (A)–(E) Ectoderm in the colored region in (A) was fated to contribute to neurons in the geniculate/anterior lateral line (aLL) ganglionic complex, and in 7/9 cases, also to the vestibuloacoustic/intracapsular ganglionic complex. (B) An embryo shortly after DiI injection at E6.5 (*t*=0) in the region shown in (A). (C) The same embryo as in (B), at E20 (*t*=14 dpi), with DiI visible in the geniculate/aLL complex. Dotted lines indicate planes of section in (D) and (E). (D) and (E) Transverse sections through (D) the geniculate/aLL ganglionic complex and (E) the vestibuloacoustic ganglion, immunostained for the neuronal marker HuC/D (green) and counterstained with DAPI (blue), showing co-localization of DiI with HuC/D. (F)–(I) Ectoderm in the colored region in (F) was fated to contribute to neurons in the petrosal ganglion. (G) An embryo shortly after DiI injection at E6.5 (*t*=0) in the region shown in (F). (H) The same embryo as in (G), at E20 (*t*=14 dpi), showing DiI in the petrosal ganglion. Dotted line indicates plane of section in (I). (I) Coronal section through the petrosal ganglion, showing co-localization of DiI and HuC/D. (J)–(N) Ectoderm in the colored region in (J) was fated to contribute to neurons in the posterior lateral line (pLL) ganglion. (K) An embryo shortly after DiI injection at E6.5 (*t*=0) in the region shown in (J). (L) The same embryo as in (K), at E20 (*t*=14 dpi), showing DiI in the pLL ganglion and the pLL nerve (inset; red arrowheads). (M) Transverse section through the petrosal and pLL ganglia showing DiI specifically in the pLL ganglion. (N) Transverse section further caudally showing DiI in the pLL nerve. (O)–(R) Ectoderm in the colored region in (O) was fated to contribute to neurons in the nodose ganglia. (P) An embryo shortly after DiI injection at E7 (*t*=0) in the region shown in (O). (Q) The same embryo as in (P), at E21 (*t*=14 dpi), showing DiI in the third and fourth nodose ganglia (arrowheads). Dotted line indicates plane of section in (R). (R) Transverse section through the fourth nodose ganglion (n4), in this case immunostained for HNK1 (green). Inset shows higher-power view of the ganglion and co-localization of DiI (red) with HNK1 immunoreactivity (green). (S) Transverse section through one of the nodose ganglia in a control embryo immunostained for HNK1 (red) and HuC/D (green), confirming expression of HNK1 in lamprey sensory ganglia. (T) Schematic summary of the fate-map for epibranchial and lateral line placode-derived ganglia at E6–7: the different regions that gave rise to neurons in the corresponding ganglia at E20–21 are indicated in varying shades of blue. Abbreviations: all, anterior lateral line; dpi, days post-injection; e, eye; g, geniculate; ICG, intracapsular ganglion; n, nodose; nt, neural tube; ov, otic vesicle; p, petrosal; pll, posterior lateral line; plln, posterior lateral line nerve; t, time; va, vestibuloacoustic. Scale bars: (B), (G), (K) and (P) 0.2 mm; (C), (H), (L) and (Q) 0.2 mm; (D), (E), (I), (M), (N), (R) and (S) 50 μm.

DiI labeling at E6–7 of cranial ectoderm caudal to the region containing opV and mmV precursors revealed the fate-map for the epibranchial and lateral line ganglia (summarized in Fig. 5A, F, J, O and T). In all embryos labeled in the area indicated in Fig. 5A (*n*=9), DiI was seen 14 days later (E20–21) in the geniculate/aLL ganglion complex rostral to the otic vesicle and also, in most cases (*n*=7/9), in the vestibuloacoustic/intracapsular ganglion complex, medial to the otic vesicle. An example of an embryo with DiI in both ganglionic complexes is shown in Fig. 5B–E (whole-mount images: Fig. 5B and C; HuC/D-immunostained transverse sections: Fig. 5D and E).

Ectoderm located caudal and ventral to geniculate/aLL-vestibuloacoustic precursors (outlined in Fig. 5F) contributed to the petrosal ganglion (*n*=3). An example is shown in Fig. 5G–I (whole-mount images: Fig. 5G and H; HuC/D-immunostained horizontal sections: Fig. 5I).

Ectoderm located dorsal to petrosal precursors and caudal to geniculate/aLL-vestibuloacoustic precursors (outlined in Fig. 5J) contributed to the pLL ganglion (*n*=8). In an example of a focal injection within this area (Fig. 5K–N), DiI was also seen in the pLL ganglion and the pLL nerve, both in whole-mount (Fig. 5L; inset) and on HuC/D-immunostained transverse sections (Fig. 5M and N).

Ectoderm located caudal to petrosal and pLL precursors (Fig. 5O) contributed to the first four nodose ganglia. In the embryo shown in Fig. 5P (labeled at E7), DiI-positive cells were observed in both the third and fourth nodose ganglia (Fig. 5Q). Transverse sections immunostained with the carbohydrate epitope antibody HNK1, which in lamprey labels cranial sensory ganglia (although it does not label cranial neural crest cells) (Hirata et al., 1997, Horigome et al., 1999), confirmed the localization of DiI-positive cells specifically within ganglia (Fig. 5R; inset). HNK1 immunoreactivity largely overlapped with HuC/D immunostaining in cranial sensory ganglia (Fig. 5S).

Our DiI labeling data also reveal regions of overlap between the precursors for different placodes at neurula stages (E6–7), shown in schematic form in Fig. 5T. Such overlap is also seen in fate-maps for gnathostome embryos at similar stages (see discussion in Pieper et al., 2011).

Taken together, these findings provide the first fate-map for placode-derived neurons in the cranial sensory ganglia of the embryonic lamprey.

### Neural crest-derived cells are found in cranial sensory ganglia

A previous *in vivo* DiI labeling study in *P. marinus* performed at E6 concluded that neural crest cells do not contribute to the cranial sensory ganglia of lampreys (McCauley and Bronner-Fraser, 2003). This was surprising, given that neural crest cells in gnathostomes form the satellite glia of all cranial sensory ganglia, plus somatosensory neurons in opV and mmV ganglia and the root ganglia of several cranial nerves (*e.g.* Yntema, 1943, Yntema, 1944, Hamburger, 1961, Narayanan and Narayanan, 1980, Ayer-Le Lièvre and Le Douarin, 1982, D'Amico-Martel and Noden, 1983, Kious et al., 2002, Harlow et al., 2011, Quina et al., 2012). Since this was a negative result, we revisited this question by labeling neural crest precursors in the dorsal neural tube at earlier neurula stages (E5–6; Piavis stage 11).

**Fig. 6:**
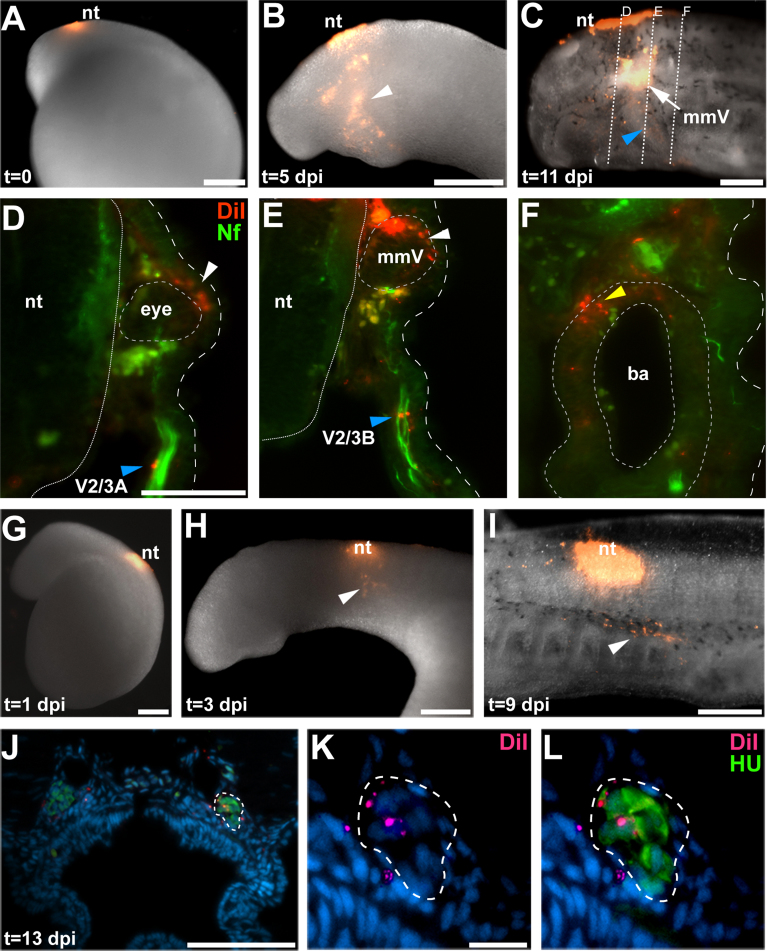
Neural crest-derived cells are found in cranial sensory ganglia and along cranial nerves (presumptive Schwann cells). (A) An E5 embryo immediately after DiI injection (*t*=0 dpi) into the presumptive rostral hindbrain. (B) At E10 (*t*=5 dpi), labeled neural crest cells are observed in optic, trigeminal and mandibular arch regions (arrowhead). (C) At E16 (*t*=11 dpi), DiI labeling is seen in the mmV ganglion (arrow) and on the lower lip/velum-innervating mmV nerve branch (V2/3B, arrowhead). Dotted lines indicate planes of section in (D)–(F). (D)–(F) In transverse sections immunostained for neurofilament (green), DiI (red) is observed in neural crest-derived cells (D) around the eye (white arrowhead) and on the upper lip-innervating mmV nerve branch (V2/3A, blue arrowhead); (E) in the mmV ganglion (white arrowhead) and on the lower lip/velum-innervating mmV nerve branch (V2/3B, blue arrowhead). (F) As expected, DiI labeling is also observed within the neural crest-derived branchial arch basket (yellow arrowhead). (G) An E6.5 embryo one day after DiI injection (*t*=1 dpi) at late E5 into the dorsal neural tube in the vagal region. (H) The same embryo as in G at E9 (*t*=3 dpi). DiI-labeled neural crest cells are observed migrating ventrally (arrowhead). (I) The same embryo at E15 (*t*=9 dpi), showing DiI-labeled neural crest cells (arrowhead) dorsal to the branchial arches. (J)–(L) At E19 (*t*=13 dpi), immunostaining on transverse sections through the nodose ganglia for the neuronal marker HuC/D (green), counterstained with DAPI (blue), revealed DiI-positive cells (red) in the nodose ganglia [(J), lower-power view; (K) and (L), higher-power views]. Abbreviations: ba, branchial arch basket; dpi, days post-injection; mmV, maxillomandibular trigeminal ganglion; nt, neural tube; t, time; V2/3A, upper lip-innervating mmV nerve branch; V2/3B, lower lip/velum-innervating mmV nerve branch. Scale bars: (A)–(C), (G)–(I) 0.2 mm; (D)–(F) and (J) 50 μm; (K) and (L) 10 μm.

DiI injection at E5 into the presumptive rostral hindbrain labeled neural crest cells that colonized the mmV ganglion (*n*=10) and peripheral nerves (presumptive Schwann cells; Nakao and Ishizawa, 1987). In an example shown in Fig. 6A–F, DiI was injected into the dorsal neural tube at early E5 (Fig. 6A). By 5 dpi (E10), neural crest cells were observed in the optic, trigeminal and mandibular arch regions (Fig. 6B). By 11 dpi (E16), DiI-positive cells were seen in the region of the mmV ganglion and along presumptive nerves (Fig. 6C). Transverse sections at different axial levels, immunostained for neurofilament, showed DiI-labeled neural crest cells around the eye and scattered on the upper lip-innervating V2/3A nerve (Fig. 6D), as well as within the mmV ganglion and scattered along the lower lip/velum-innervating V2/3B nerve (Fig. 6E). DiI-positive cells were also observed in the cartilage of the branchial baskets, confirming successful neural crest labeling (Fig. 6F).

DiI injection at E5–6 into the dorsal neural tube in the vagal region revealed that neural crest cells colonize the nodose ganglia (*n*=7). In an example shown in Fig. 6G–L, at 1 dpi (E7), DiI-labeled cells were still largely restricted to the dorsal neural tube (Fig. 6G). However, by 3 dpi (E8–9), DiI-positive neural crest cells were observed ventral to the neural tube (Fig. 6H), while by 9 dpi (E14), they were found dorsal to the developing branchial arches (Fig. 6I). By 13 dpi (E18–19), HuC/D immunostaining on transverse sections revealed DiI-positive cells in nodose ganglia (Fig. 6J–L). Taken together, these results demonstrate that neural crest cells colonize cranial sensory ganglia (and give rise to presumptive glial cells) in agnathans.

## Discussion

In recent years, the key transcription factors and signaling pathways involved in patterning the preplacodal region and neurogenic placodes have been elucidated in representative gnathostomes (chick, mouse, *Xenopus*, zebrafish; reviewed in Streit, 2007, Ladher et al., 2010, Schlosser, 2010, Grocott et al., 2012). Molecular developmental studies in lampreys help shed light on the developmental processes and mechanisms that are shared between agnathans and gnathostomes, hence likely to have been inherited from the vertebrate ancestor (see Osório and Rétaux, 2008, Shimeld and Donoghue, 2012). Several studies have described neurogenic placode, cranial sensory ganglion and nerve development in different lamprey species, based on histology (e.g. von Kupffer, 1891, von Kupffer, 1895, Damas, 1944, Fisk, 1954) or whole-mount axonal immunostaining (Kuratani et al., 1997, Kuratani et al., 1998, Barreiro-Iglesias et al., 2008). Here, we DiI-labeled different regions of late neurula-stage cranial ectoderm to provide the first detailed fate-map for placode-derived neurons in cranial sensory ganglia in the sea lamprey *P. marinus*. This was coupled with immunostaining for neuronal markers to describe the precise spatiotemporal development of cranial sensory ganglia.

Based on our findings, we define key stages of neurogenic placode development in *P. marinus*. Our fate-map suggests that at E6–7, the precursors for ophthalmic trigeminal (opV) neurons and both groups of maxillomandibular (mmV) neurons, *i.e.*, upper lip-innervating V2/3A neurons and lower lip/velum-innervating V2/3B neurons (Oisi et al., 2013), are already largely separable (albeit with some overlap between opV and presumptive V2/3B neuronal precursors; see next section). In contrast, the more caudally located precursors for epibranchial, otic and lateral line placode-derived neurons still show extensive overlap at this stage, suggesting that individual placode specification from a larger common placode field is ongoing (as seen in similar early-stage fate-maps in chick and *Xenopus*; Streit, 2002, Xu et al., 2008, Pieper et al., 2011). Therefore, we hypothesize that the preplacodal region is being established during E4–5 (late gastrula–early neurula), followed by an extended period of segregation of individual placodes from a larger common field from E5–6 to E8–9. Immunostaining using a cross-reactive Pax3/7 antibody shows that the pan-vertebrate ophthalmic trigeminal (opV/profundal) marker Pax3 (lamprey Pax3/7: this study; gnathostome Pax3: Stark et al., 1997, Baker et al., 1999, Schlosser and Ahrens, 2004, O'Neill et al., 2007, Modrell et al., 2011) and the neuronal marker HuC/D first begin to be expressed in the developing ganglia from E8. Thus, there is rapid progression to neurogenesis upon placode formation, with ganglion formation well underway by E10, suggesting that the key stages for the regulation of placode-derived neuron differentiation are E7–10.

### Spatial segregation of lamprey mmV neuronal precursors may prefigure later spatial segregation and somatotopy within the lamprey mmV ganglion

The somatotopy of lamprey mmV nerve projections is reflected by the spatial segregation of different afferents within the mmV ganglion: lower lip/velum-innervating (V2/3B) neurons are found in the rostral part of the ganglion, while upper lip-innervating (V2/3A) neurons are located in the caudal part of the ganglion (Koyama et al., 1987, Kuratani et al., 2004, Murakami and Kuratani, 2008). Intriguingly, our fate-map suggests that their precursors are similarly spatially segregated in two discrete patches at late neurula stages, with V2/3B precursors located rostral to V2/3A precursors, and opV neuron precursors sandwiched between them. We have tentatively identified the anteroventral pool of mmV neuron precursors (rostral to but partially overlapping with opV neuron precursors) as lower lip/velum-innervating V2/3B neuron precursors, because the neurons formed by this pool are confined to a small, rostral domain of the mmV ganglion, which seems to correspond to the location of lower lip/velum afferents as defined by dextran–biotin nerve-tracing in *Lethenteron camtschaticum* (*Lethenteron japonicum*) (Kuratani et al., 2004, Murakami and Kuratani, 2008). Similarly, we have tentatively identified the caudal patch of mmV neuron precursors at neurula stages (caudal to opV neuron precursors) as upper lip-innervating V2/3A precursors, because the neurons formed by this pool are confined to a larger, caudal domain of the mmV ganglion that seems to correspond to the location of upper lip afferents (Kuratani et al., 2004, Murakami and Kuratani, 2008). If this interpretation is correct, the somatotopy of the lamprey mmV ganglion may reflect a very early developmental distinction between neurons destined to innervate the upper lip versus the lower lip/velum, and perhaps even the induction of separate placodes for these neurons.

Previous histological studies in lamprey embryos have described morphologically identifiable opV and mmV placodes, with the opV placode located immediately rostrally to the mmV placode (von Kupffer, 1895, Damas, 1944, Fisk, 1954). Hence, whether or not these two pools of mmV neuron precursors represent distinct V2/3A and V2/3B placode precursors, differential growth, and/or morphological movements associated with optic cup evagination, must bring these two patches of ectoderm together, caudal to the patch of opV placode precursors, to form the mmV placode identified morphologically in previous studies (von Kupffer, 1895, Damas, 1944, Fisk, 1954). In *Xenopus*, mmV placode precursors (which in this species express Pax6; Schlosser and Ahrens, 2004) initially lie rostral to opV placode precursors (Pieper et al., 2011), in contrast to the situation in chick (Xu et al., 2008). In *Xenopus*, unlike in chick, both opV and mmV placodes develop relatively close to the optic cups: it has been proposed that morphological movements associated with eye evagination displace the lateral part of the rostral-most preplacodal ectoderm ventrocaudally, such that after neural tube closure, the mmV placode is induced ventral and caudal to the opV placode (Pieper et al., 2011).

Although the spatial segregation of mmV neuron precursors may of course be a derived feature of lampreys, the mmV nerve in gnathostomes also exhibits somatotopy, with maxillary and mandibular neurons spatially segregated in the mmV ganglion. Nerve-tracing experiments have shown that maxillary and mandibular neurons are physically separated within the snake mmV ganglion by a septum of connective tissue and blood vessels (Molenaar, 1978). Maxillary and mandibular neurons are also spatially segregated within the maxillomandibular lobe of the trigeminal ganglion in birds, mammals and turtles, albeit with some overlap (Dubbeldam and Veenman, 1978, Noden, 1980a, Noden, 1980b, Erzurumlu and Jhaveri, 1992, Rhinn et al., 2013) and in the teleost trigeminal ganglion (Kerem et al., 2005). (For a helpful pictorial overview of the segregation of opV/profundal, maxillary and mandibular trigeminal neurons in various vertebrate groups, see Fig. 2 in Kerem et al., 2005). In mouse, the spatial segregation of maxillary and mandibular neurons is established before axon outgrowth, *i.e.*, before any contact with peripheral targets (Erzurumlu and Jhaveri, 1992, Scott and Atkinson, 1999, Hodge et al., 2007). Indeed, expression of the transcription factor Hmx1 is restricted to neurons in the mandibular (caudal-most) portion of the mouse trigeminal ganglion as early as E9.5 (Hodge et al., 2007) (also see Erzurumlu et al., 2010). Moreover, existing fate-map data cannot rule out the possibility that maxillary and mandibular trigeminal neuron precursors are spatially segregated within the mmV placode in gnathostomes. The recent detailed fate-map of *Xenopus* neurogenic placodes did not track labeled cells through to ganglion stages (Pieper et al., 2011). In chick, where ganglion stages were examined, the quail–chick grafting approach was probably insufficiently fine-grained (D'Amico-Martel and Noden, 1983), while after focal DiI labeling, it would have been very difficult to distinguish maxillary versus mandibular neurons on transverse sections without any markers, even had the possibility of spatial segregation been considered when these experiments were performed (Xu et al., 2008). Whether the gnathostome mmV placode is in fact bipartite, with segregated precursors for maxillary and mandibular trigeminal neurons, remains an intriguing possibility for future research. Unfortunately, no cross-species molecular markers for mmV placode cells have as yet been identified, in contrast to opV placode cells, which express Pax3 in all gnathostomes (Stark et al., 1997, Baker et al., 1999, Schlosser and Ahrens, 2004, O'Neill et al., 2007, Modrell et al., 2011). As shown here by immunostaining with a pan-Pax3/7 antibody (Davis et al., 2005), lamprey opV neurons also express the single lamprey *Pax3/7* gene (McCauley and Bronner-Fraser, 2002, Osório et al., 2005, Kusakabe et al., 2011), confirming Pax3 as a pan-vertebrate marker for opV placode-derived neurons.

### Lamprey cranial sensory ganglia contain neural crest-derived cells

A previous fate-mapping study in *P. marinus* in which premigratory neural crest cells were DiI-labeled at approximately E6 demonstrated a neural crest contribution to the branchial arches but not to the cranial sensory ganglia, unless ectoderm was also labeled (McCauley and Bronner-Fraser, 2003). Furthermore, the expression of a lamprey homolog of *Sox10*, which in gnathostomes is expressed in migrating neural crest cells and maintained in the peripheral glial lineage (see Britsch et al., 2001), was excluded from developing cranial ganglia (McCauley and Bronner-Fraser, 2003). The authors suggested that the neural crest contribution to the cranial sensory ganglia may have arisen within the gnathostome lineage. This is surprising because in gnathostomes, cranial neural crest cells not only give rise to somatosensory neurons in the trigeminal ganglia and proximal (“root”) ganglia of other cranial nerves, but also to the satellite glia of all cranial sensory ganglia (*e.g.* Yntema, 1943, Yntema, 1944, Hamburger, 1961, Narayanan and Narayanan, 1980, Ayer-Le Lièvre and Le Douarin, 1982, D'Amico-Martel and Noden, 1983, Kious et al., 2002, Harlow et al., 2011, Quina et al., 2012). A neural crest contribution to lamprey cranial sensory ganglia is supported by their reduction in lamprey embryos in which the function of various neural crest specifier genes (*e.g.* from the *Msx*, *Zic*, *Id* and *FoxD3* gene families) was knocked down using anti-sense morpholinos (Sauka-Spengler et al., 2007). Here, we labeled cranial neural crest cell precursors at E5–6 (up to 24 h earlier than McCauley and Bronner-Fraser, 2003) and observed DiI-positive cells migrating away from the injection site to contribute to several cranial sensory ganglia, including the mmV and nodose ganglia, as well as scattered cells along their nerve fibers (presumptive Schwann cells; Nakao and Ishizawa, 1987). We suggest that differences in the time of injection may explain the lack of DiI-labeled neural crest cells in the cranial sensory ganglia in previous fate-mapping experiments (McCauley and Bronner-Fraser, 2003). Overall, we conclude that the mechanisms underlying the development of neurogenic placodes and cranial sensory ganglia in the lamprey are likely to be highly conserved with gnathostomes.

## Footnotes

Supplementary material associated with this article can be found in the online version at doi:10.1016/j.ydbio.2013.10.021.

## References

[bib1] Ayer-Le Lièvre C.S., Le Douarin N.M. (1982). The early development of cranial sensory ganglia and the potentialities of their component cells studied in quail–chick chimeras. Dev. Biol..

[bib2] Baker C.V.H., Stark M.R., Marcelle C., Bronner-Fraser M. (1999). Competence, specification and induction of Pax-3 in the trigeminal placode. Development.

[bib3] Barreiro-Iglesias A., Gómez-López M.P., Anadón R., Rodicio M.C. (2008). Early development of the cranial nerves in a primitive vertebrate, the sea lamprey, *Petromyzon marinus* L.. Open Zool. J..

[bib4] Britsch S., Goerich D.E., Riethmacher D., Peirano R.I., Rossner M., Nave K.A., Birchmeier C., Wegner M. (2001). The transcription factor Sox10 is a key regulator of peripheral glial development. Genes Dev..

[bib5] Curran K., Lister J.A., Kunkel G.R., Prendergast A., Parichy D.M., Raible D.W. (2010). Interplay between Foxd3 and Mitf regulates cell fate plasticity in the zebrafish neural crest. Dev. Biol..

[bib6] D'Amico-Martel A. (1982). Temporal patterns of neurogenesis in avian cranial sensory and autonomic ganglia. Am. J. Anat..

[bib7] D'Amico-Martel A., Noden D.M. (1983). Contributions of placodal and neural crest cells to avian cranial peripheral ganglia. Am. J. Anat..

[bib8] Damas H. (1944). Recherches sur le développement de *Lampetra fluviatilis* L. Contribution à l'étude de la céphalogenèse des vertébrés. Arch. Biol..

[bib9] Davis G.K., D'Alessio J.A., Patel N.H. (2005). Pax3/7 genes reveal conservation and divergence in the arthropod segmentation hierarchy. Dev. Biol..

[bib10] Dubbeldam J.L., Veenman C.L. (1978). Studies on the somatotopy of the trigeminal system in the mallard, *Anas platyrhynchos* L.: I. The ganglion trigeminale. Neth. J. Zool..

[bib11] Dude C.M., Kuan C.-Y.K., Bradshaw J.R., Greene N.D.E., Relaix F., Stark M.R., Baker C.V.H. (2009). Activation of Pax3 target genes is necessary but not sufficient for neurogenesis in the ophthalmic trigeminal placode. Dev. Biol..

[bib12] Erzurumlu R.S., Jhaveri S. (1992). Trigeminal ganglion cell processes are spatially ordered prior to the differentiation of the vibrissa pad. J. Neurosci..

[bib13] Erzurumlu R.S., Murakami Y., Rijli F.M. (2010). Mapping the face in the somatosensory brainstem. Nat. Rev. Neurosci..

[bib14] Fisk A. (1954). The early development of the ear and acoustico–facialis complex of ganglia in the Lamprey *Lampetra planeri* Bloch. Proc. Zool. Soc. Lond..

[bib15] Graham A., Shimeld S.M. (2013). The origin and evolution of the ectodermal placodes. J. Anat..

[bib16] Grocott T., Tambalo M., Streit A. (2012). The peripheral sensory nervous system in the vertebrate head: a gene regulatory perspective. Dev. Biol..

[bib17] Hall B.K., Gillis J.A. (2013). Incremental evolution of the neural crest, neural crest cells and neural crest-derived skeletal tissues. J. Anat..

[bib18] Hamburger V. (1961). Experimental analysis of the dual origin of the trigeminal ganglion in the chick embryo. J. Exp. Zool..

[bib19] Harlow D.E., Yang H., Williams T., Barlow L.A. (2011). Epibranchial placode-derived neurons produce BDNF required for early sensory neuron development. Dev. Dyn..

[bib20] Hinman M.N., Lou H. (2008). Diverse molecular functions of Hu proteins. Cell. Mol. Life Sci..

[bib21] Hirata M., Ito K., Tsuneki K. (1997). Migration and colonization patterns of HNK-1-immunoreactive neural crest cells in lamprey and swordtail embryos. Zool. Sci..

[bib22] Hodge L.K., Klassen M.P., Han B.X., Yiu G., Hurrell J., Howell A., Rousseau G., Lemaigre F., Tessier-Lavigne M., Wang F. (2007). Retrograde BMP signaling regulates trigeminal sensory neuron identities and the formation of precise face maps. Neuron.

[bib23] Horigome N., Myojin M., Ueki T., Hirano S., Aizawa S., Kuratani S. (1999). Development of cephalic neural crest cells in embryos of *Lampetra japonica*, with special reference to the evolution of the jaw. Dev. Biol..

[bib24] Kerem G., Yoshimoto M., Yamamoto N., Yang C.Y., Xue H.G., Ito H. (2005). Somatotopic organization of the trigeminal ganglion cells in a cichlid fish, *Oreochromis (Tilapia) niloticus*. Brain Behav. Evol..

[bib25] Kious B.M., Baker C.V.H., Bronner-Fraser M., Knecht A.K. (2002). Identification and characterization of a calcium channel gamma subunit expressed in differentiating neurons and myoblasts. Dev. Biol..

[bib26] Koyama H., Kishida R., Goris R.C., Kusunoki T. (1987). Organization of sensory and motor nuclei of the trigeminal nerve in lampreys. J. Comp. Neurol..

[bib27] Koyama H., Kishida R., Goris R.C., Kusunoki T. (1990). Organization of the primary projections of the lateral line nerves in the lamprey *Lampetra japonica*. J. Comp. Neurol..

[bib28] Kuratani S., Murakami Y., Nobusada Y., Kusakabe R., Hirano S. (2004). Developmental fate of the mandibular mesoderm in the lamprey, *Lethenteron japonicum*: comparative morphology and development of the gnathostome jaw with special reference to the nature of the *Trabecula cranii*. J. Exp. Zool. B: Mol. Dev. Evol..

[bib29] Kuratani S., Horigome N., Ueki T., Aizawa S., Hirano S. (1998). Stereotyped axonal bundle formation and neuromeric patterns in embryos of a cyclostomes *Lampetra japonica*. J. Comp. Neurol..

[bib30] Kuratani S., Ueki T., Aizawa S., Hirano S. (1997). Peripheral development of cranial nerves in a cyclostome, *Lampetra japonica*: morphological distribution of nerve branches and the vertebrate body plan. J. Comp. Neurol..

[bib31] Kusakabe R., Kuraku S., Kuratani S. (2011). Expression and interaction of muscle-related genes in the lamprey imply the evolutionary scenario for vertebrate skeletal muscle, in association with the acquisition of the neck and fins. Dev. Biol..

[bib32] Ladher R.K., O'Neill P., Begbie J. (2010). From shared lineage to distinct functions: the development of the inner ear and epibranchial placodes. Development.

[bib33] McCauley D.W., Bronner-Fraser M. (2002). Conservation of *Pax* gene expression in ectodermal placodes of the lamprey. Gene.

[bib34] McCauley D.W., Bronner-Fraser M. (2003). Neural crest contributions to the lamprey head. Development.

[bib35] Minchin J.E., Hughes S.M. (2008). Sequential actions of Pax3 and Pax7 drive xanthophore development in zebrafish neural crest. Dev. Biol..

[bib36] Modrell M.S., Buckley D., Baker C.V.H. (2011). Molecular analysis of neurogenic placode development in a basal ray-finned fish. Genesis.

[bib37] Molenaar G.J. (1978). The sensory trigeminal system of a snake in the possession of infrared receptors. I. The sensory trigeminal nuclei. J. Comp. Neurol..

[bib38] Murakami Y., Kuratani S. (2008). Brain segmentation and trigeminal projections in the lamprey; with reference to vertebrate brain evolution. Brain Res. Bull..

[bib39] Murakami Y., Watanabe A. (2009). Development of the central and peripheral nervous systems in the lamprey. Dev. Growth Differ..

[bib40] Murakami Y., Ogasawara M., Sugahara F., Hirano S., Satoh N., Kuratani S. (2001). Identification and expression of the lamprey *Pax6* gene: evolutionary origin of the segmented brain of vertebrates. Development.

[bib41] Nakao T., Ishizawa A. (1987). Development of the spinal nerves of the larval lamprey: IV. Spinal nerve roots of 21-mm larval and adult lampreys, with special reference to the relation of meninges with the root sheath and the perineurium. J. Comp. Neurol..

[bib42] Narayanan C.H., Narayanan Y. (1980). Neural crest and placodal contributions in the development of the glossopharyngeal–vagal complex in the chick. Anat. Rec..

[bib43] Neidert A.H., Virupannavar V., Hooker G.W., Langeland J.A. (2001). Lamprey *Dlx* genes and early vertebrate evolution. Proc. Natl. Acad. Sci. U.S.A.

[bib44] Nikitina, N., Bronner-Fraser, M., Sauka-Spengler, T., 2009. The Sea Lamprey *Petromyzon marinus*: A Model for Evolutionary and Developmental Biology. CSH Protoc. 2009, pdb.emo113. 10.1101/pdb.emo113 20147008

[bib45] Nikitina N., Sauka-Spengler T., Bronner-Fraser M. (2008). Dissecting early regulatory relationships in the lamprey neural crest gene network. Proc. Natl. Acad. Sci. U.S.A.

[bib46] Noden D.M. (1980). Somatotopic and functional organization of the avian trigeminal ganglion: an HRP analysis in the hatchling chick. J. Comp. Neurol..

[bib47] Noden D.M. (1980). Somatotopic organization of the embryonic chick trigeminal ganglion. J. Comp. Neurol..

[bib48] Northcutt R.G. (1979). Experimental determination of the primary trigeminal projections in lampreys. Brain Res..

[bib49] O'Neill P., McCole R.B., Baker C.V.H. (2007). A molecular analysis of neurogenic placode and cranial sensory ganglion development in the shark, *Scyliorhinus canicula*. Dev. Biol..

[bib50] Oisi Y., Ota K.G., Kuraku S., Fujimoto S., Kuratani S. (2013). Craniofacial development of hagfishes and the evolution of vertebrates. Nature.

[bib51] Osório J., Rétaux S. (2008). The lamprey in evolutionary studies. Dev. Genes Evol..

[bib52] Osório J., Mazan S., Rétaux S. (2005). Organisation of the lamprey (*Lampetra fluviatilis*) embryonic brain: insights from LIM-homeodomain, Pax and hedgehog genes. Dev. Biol..

[bib53] Ota K.G., Kuraku S., Kuratani S. (2007). Hagfish embryology with reference to the evolution of the neural crest. Nature.

[bib54] Patel N.H. (1994). Imaging neuronal subsets and other cell types in whole-mount *Drosophila* embryos and larvae using antibody probes. Methods Cell Biol..

[bib55] Piavis G.W. (1961). Embryological stages in the sea lamprey and effects of temperature on development. Fish. Bull. Fish Wildl. Serv. U.S..

[bib56] Pieper M., Eagleson G.W., Wosniok W., Schlosser G. (2011). Origin and segregation of cranial placodes in *Xenopus laevis*. Dev. Biol..

[bib57] Prasad M.S., Sauka-Spengler T., LaBonne C. (2012). Induction of the neural crest state: control of stem cell attributes by gene regulatory, post-transcriptional and epigenetic interactions. Dev. Biol..

[bib58] Quina L.A., Tempest L., Hsu Y.-W., Cox T.C., Turner E.E. (2012). Hmx1 is required for the normal development of somatosensory neurons in the geniculate ganglion. Dev. Biol..

[bib59] Rhinn M., Miyoshi K., Watanabe A., Kawaguchi M., Ito F., Baker C.V.H., Murakami Y., Rijli F.M. (2013). Evolutionary divergence of trigeminal nerve somatotopy in amniotes. J. Comp. Neurol..

[bib60] Richardson M.K., Wright G.M. (2003). Developmental transformations in a normal series of embryos of the sea lamprey *Petromyzon marinus* (Linnaeus). J. Morphol..

[bib61] Sauka-Spengler T., Bronner-Fraser M. (2008). Insights from a sea lamprey into the evolution of neural crest gene regulatory network. Biol. Bull..

[bib62] Sauka-Spengler T., Meulemans D., Jones M., Bronner-Fraser M. (2007). Ancient evolutionary origin of the neural crest gene regulatory network. Dev. Cell.

[bib63] Schlosser G. (2010). Making senses: development of vertebrate cranial placodes. Int. Rev. Cell Mol. Biol..

[bib64] Schlosser G. (2006). Induction and specification of cranial placodes. Dev. Biol..

[bib65] Schlosser G., Ahrens K. (2004). Molecular anatomy of placode development in *Xenopus laevis*. Dev. Biol..

[bib66] Scott L., Atkinson M.E. (1999). Compartmentalisation of the developing trigeminal ganglion into maxillary and mandibular divisions does not depend on target contact. J. Anat..

[bib67] Shigetani Y., Sugahara F., Kawakami Y., Murakami Y., Hirano S., Kuratani S. (2002). Heterotopic shift of epithelial–mesenchymal interactions in vertebrate jaw evolution. Science.

[bib68] Shimeld S.M., Donoghue P.C.J. (2012). Evolutionary crossroads in developmental biology: cyclostomes (lamprey and hagfish). Development.

[bib69] Somorjai I.M., Somorjai R.L., Garcia-Fernàndez J., Escrivà H. (2012). Vertebrate-like regeneration in the invertebrate chordate amphioxus. Proc. Natl. Acad. Sci. U.S.A..

[bib70] Stark M.R., Sechrist J., Bronner-Fraser M., Marcelle C. (1997). Neural tube–ectoderm interactions are required for trigeminal placode formation. Development.

[bib71] Streit A. (2007). The preplacodal region: an ectodermal domain with multipotential progenitors that contribute to sense organs and cranial sensory ganglia. Int. J. Dev. Biol..

[bib72] Streit A. (2002). Extensive cell movements accompany formation of the otic placode. Dev. Biol..

[bib73] von Kupffer C. (1891). The development of the cranial nerves of vertebrates. J. Comp. Neurol..

[bib74] von Kupffer, C., 1895. Studien zur Vergleichenden Entwicklungsgeschichte des Kopfes der Kranioten. III. Die Entwicklung der Kopfnerven von *Ammocoetes planeri*. Munich: J.F. Lehmann.

[bib75] Wang, Q., Arighi, C.N., King, B.L., Polson, S.W., Vincent, J., Chen, C., Huang, H., Kingham, B.F., Page, S.T., Rendino, M.F., Thomas, W.K., Udwary, D.W., Wu, C.H., North East Bioinformatics Collaborative Curation Team, 2012. Community annotation and bioinformatics workforce development in concert - little skate genome annotation workshops and jamborees. Database (Oxford) 2012, bar064. 10.1093/database/bar064 PMC3308154 22434832

[bib76] Wicht H., Northcutt R.G. (1995). Ontogeny of the head of the Pacific hagfish (*Eptatretus stouti*, Myxinoidea): development of the lateral line system. Philos. Trans. R. Soc. Lond. B.

[bib77] Xu H., Dude C.M., Baker C.V.H. (2008). Fine-grained fate maps for the ophthalmic and maxillomandibular trigeminal placodes in the chick embryo. Dev. Biol..

[bib78] Yntema C.L. (1943). An experimental study on the origin of the sensory neurones and sheath cells of the IXth and Xth cranial nerves in *Amblystoma punctatum*. J. Exp. Zool..

[bib79] Yntema C.L. (1944). Experiments on the origin of the sensory ganglia of the facial nerve in the chick. J. Comp. Neurol..

