# Supplementary material for: A fate-map for cranial sensory ganglia in the sea lamprey
Source: Dev Biol. 2014 Jan 15;385(2):405–16. doi: 10.1016/j.ydbio.2013.10.021 (PMC3928997; doi:10.1016/j.ydbio.2013.10.021)
Supplement: Supplementary file 1 — Supplementary material [file mmc1.doc]

**Supplemental Methods**

**Table 1:** List of species including common names for both ingroup and chosen outgroups with GenBank accession numbers, Ensembl protein codes or SkateBase (www.skatebase.org) contig identifiers. N/A=not available.

| **Species name** | **Common name** | **Pax3/7** | **Pax3** | **Pax7** |
| --- | --- | --- | --- | --- |
| *Branchiostoma belcheri* | Belcher's lancelet | ABK54280.1 |  |  |
| *Branchiostoma floridae* | Florida lancelet | EEN66816.1 |  |  |
| *Ciona intestinalis* | yellow seasquirt (vase tunicate) | XP_002122162.2 |  |  |
| *Danio rerio* | zebrafish |  | AAC41253.1 | CAM12909.1 |
| *Eptatretus burgeri* | inshore hagfish | BAG11537.1 |  |  |
| *Gallus gallus* | chicken |  | BAB85652.1 | NP_990396.1 |
| *Homo sapiens* | human |  | AAI14364.1 | CAA65522.1 |
| *Lampetra fluviatilis* | river lamprey | AAY90105.1 |  |  |
| *Latimeria chalumnae* | coelacanth |  | ENSLACP00000007147 | ENSLACP00000021802 |
| *Lethenteron camtschaticum* | Arctic lamprey | ADP37890.1 |  |  |
| *Leucoraja erinacea* | little skate |  | Contig10764 | Contig14797 |
| *Mus musculus* | house mouse |  | AAH48699.1 | AAG16663.3 |
| *Oryzias latipes* | Japanese rice fish (medaka) |  | XP_004079275.1 | XP_004068688.1 |
| *Petromyzon marinus* | sea lamprey | AAL04156.1 |  |  |
| *Polyodon spathula* | Mississippi paddlefish |  | ADZ48385.1 | N/A |
| *Scyliorhinus canicula* | small-spotted catshark (lesser-spotted dogfish) |  | ABM89502.1 | Contigs30938, 19995 |
| *Scyliorhinus torazame* | cloudy catshark |  | BAM15710.1 | BAM15711.1 |
| *Takifugu rubripes* | Japanese pufferfish |  | ENSTRUP00000023136 | ENSTRUP00000038950 |
| *Xenopus laevis* | African clawed frog |  | AAV31937.1 | NP_001088995.1 |
| *Xiphophorus maculatus* | Southern platyfish |  | ENSXMAP00000015307 | ENSXMAP00000012910 |

**Phylogenetic analyses**

To analyze the phylogenetic relationships of the Pax3/7 subfamily of transcription factors in chordates, we conducted a phylogenetic analysis primarily using sequences available from GenBank (National Center for Biotechnology Information). Sequences for *Takifugu rubripes*, *Latimeria chalumnae* and *Xiphophorus maculatus* were obtained from the Ensembl database (www.ensembl.org), while sequences for *Leucoraja erinacea* and *Scyliorhinus canicula* (Pax7 only) were obtained from SkateBase (www.skatebase.org). Several factors can hamper establishing robust phylogenetic hypotheses in these kinds of analyses. Taxonomic sampling is usually fairly incomplete, normally consisting of single representatives of evolutionarily distant lineages (e.g., *Gallus*, *Mus*, *Homo*, *Danio* and *Xenopus* to represent all vertebrate diversity). Sequences used are normally short, and heterogeneous in composition, containing both highly conserved and highly variable regions, which makes retrieving a clear phylogenetic signal difficult. Moreover, sequences are troublesome to align, and the resulting alignment matrices generally contain many ambiguous regions and gaps. Furthermore, finding suitable outgroups to root phylogenetic hypotheses appropriately is also problematic given the highly divergent nature of the ingroup sequences. To deal with these potential problems, we followed several different approaches.

First, we aligned the amino acids data matrix in MAFFT (Katoh, 2013), refining the resulting alignment by eye in Mesquite (Maddison and Maddison, 2011) by deleting ambiguous positions that could not be aligned properly. This data matrix was analyzed under a Bayesian framework in MrBayes 3.2.1 (Ronquist et al., 2012). As a prior for the amino acids substitution model, we used the option “aamodelpr=mixed”, allowing the Bayesian search to explore all possible fixed substitution matrices. We ran two different analyses for 10x10^6^ generations, sampling every 1000 generations. We checked for convergence and appropriate mixing of the MCMC chains in Tracer v1.5 (available at tree.bio.ed.ac.uk/software/tracer/). To analyze the impact of outgroup selection, we compared the results obtained in the Bayesian analysis with those obtained under a coalescence approach as implemented in the software BEAST 1.7.5 (Drummond et al., 2012). This approach is based on the use of a population mathematical model, the coalescent, together with different implemented molecular clocks, to yield rooted phylogenetic hypotheses with the root representing the coalescence time/position of all the genetic variants observed at the tips of the tree. Outgroups, thus, are not strictly necessary in this kind of analyses to root the tree. We ran analyses with and without outgroups. We specified a relaxed molecular clock (uncorrelated lognormal, mean=1) as a prior for the substitution rates, together with a ‘birth-and-death process with incomplete sampling’ as a prior for the topology of the tree. We ran the analyses for 10x10^6^ generations, sampling every 1000 generations. Again, we checked for convergence and appropriate mixing of the MCMC chains in Tracer v1.5. The resulting posterior distribution of trees was summarized in a Maximum Clade Credibility tree (MCC) in TreeAnnotator v1.7.5 (part of the BEAST software package), representing the tree with the maximum sum of posterior probabilities. MrBayes 3.2.1 and BEAST 1.7.5 analyses were run in the online CIPRES Science Gateway (Miller et al., 2010).

Second, we followed an ‘alignment free’ approach, as implemented in the software BAli-Phy (Redelings and Suchard, 2005; Suchard and Redelings, 2006). Generally, the first step in any phylogenetic analyses is to establish a fixed alignment that represents the hypotheses of homology among the positions of the gene and taxa. The alignment, however, may lead to incorrect phylogenetic hypotheses if it contains many ambiguous/misaligned regions. Basically, BAli-Phy follows a Bayesian-iterative approach, considering the alignment as another parameter of the analysis, which is estimated together with all the other parameters, e.g., branch lengths and topology, during the analysis. Bali-Phy explores the ambiguities, and considers all the ‘near to the optimum’ possible alignments and their associated phylogenies. Importantly, this method takes into account the information on shared ‘indels’ to help reconstructing the phylogenetic trees. The program returns a phylogenetic hypothesis and the alignment that generated it, together with the uncertainty associated to the alignment, and the assessment of confidence on both the phylogeny and the alignment using posterior probabilities. Phylogenetic trees were edited in FigTree v1.4.0 (http://tree.bio.ed.ac.uk/software/figtree/).

**Supplemental References:**

Drummond AJ, Suchard MA, Xie D, Rambaut A. 2012. Bayesian phylogenetics with BEAUti and the BEAST 1.7. *Mol. Biol. Evol*. 29: 1969-1973.

Katoh S. 2013. MAFFT multiple sequence alignment software version 7: improvements in performance and usability. *Mol. Biol. Evol*. 30: 772-780.

Maddison WP, Maddison DR. 2011. Mesquite: a modular system for evolutionary analysis. Version 2.75 [http://mesquiteproject.org](http://mesquiteproject.org/)

Miller MA, Pfeiffer W, Schwartz T. 2010. Creating the CIPRES Science Gateway for inference of large phylogenetic trees. In: Proceedings of the Gateway Computing Environment Workshop (GCE), 14 Nov. 2010. New Orleans, LA, pp 1-8.

Redelings BD, Suchard MA. 2005. Joint Bayesian estimation of alignment and phylogeny. *Syst. Biol*. 54: 401-418.

Ronquist F, Teslenko M, van der Mark P, Ayres DL, Darling A, Hohna S, Larget B, Liu L, Suchard MA, Huelsenbeck JP. 2012. MrBayes 3.2: Efficient Bayesian phylogenetic inference and model choice across a large model space. *Syst. Biol*. 61: 539-542.

Suchard MA, Redelings BD. 2006. Bali-Phy: simultaneous Bayesian inference of alignment and phylogeny. *Bioinformatics* 22: 2047-2048.
